# Headache onset after vaccination against SARS-CoV-2: a systematic literature review and meta-analysis

**DOI:** 10.1186/s10194-022-01400-4

**Published:** 2022-03-31

**Authors:** Matteo Castaldo, Marta Waliszewska-Prosół, Maria Koutsokera, Micaela Robotti, Marcin Straburzyński, Loukia Apostolakopoulou, Mariarita Capizzi, Oneda Çibuku, Fidel Dominique Festin Ambat, Ilaria Frattale, Zukhra Gadzhieva, Erica Gallo, Anna Gryglas-Dworak, Gleni Halili, Asel Jusupova, Yana Koperskaya, Alo-Rainer Leheste, Maria Laura Manzo, Andrea Marcinnò, Antonio Marino, Petr Mikulenka, Bee Eng Ong, Burcu Polat, Zvonimir Popovic, Eduardo Rivera-Mancilla, Adina Maria Roceanu, Eleonora Rollo, Marina Romozzi, Claudia Ruscitto, Fabrizio Scotto di Clemente, Sebastian Strauss, Valentina Taranta, Maria Terhart, Iryna Tychenko, Simone Vigneri, Blazej Misiak, Paolo Martelletti, Alberto Raggi, On behalf of the European Headache Federation School of Advanced Studies (EHF-SAS)

**Affiliations:** 1 CNAP, Center for Sensory-Motor Interaction (SMI), Department of Health Science and Technology, Faculty of Medicine, Aalborg University, Aalborg, Denmark; 2 Department of Neurology, Wroclaw Medical University, Wroclaw, Poland; 3 “Thriassio” General Hospital of Elefsina, Elefsina, Greece; 4 Ambulatorio Per La Diagnosi E Cura Delle Cefalee, Centro Medico Visconti Di Modrone, Milan, Italy; 5 Headache Clinic, Terapia Neurologiczna ‘Samodzielni’, Warsaw, Poland; 6 General Practice, Orzyny, Poland; 7 First Neurology Department, Medical School, Eginition University Hospital, National and Kapodistrian University of Athens, Athens, Greece; 8 Department of Health Promotion, Mother and Child Care, Internal Medicine and Medical Specialties, Policlinico Universitario “P. Giaccone”, University of Palermo, Palermo, Italy; 9 Outpatient Service, Pogradec Hospital, Pogradec, Albania; 10 Deparment of Internal Medicine, Sapienza University, Rome, Italy; 11 Child Neurology and Psychiatry Unit, Systems Medicine Department, Tor Vergata University Hospital of Rome, Rome, Italy; 12 Neurology Department, Republican Diagnostic Centre, Makhachkala, Russia; 13 3Rd Neurology Department of Cerebrovascular Diseases and Cognitive Impairments, Research Center of Neurology, Moscow, Russia; 14 Department of Neurosciences “Rita Levi Montalcini”, University of Torino, Turin, Italy; 15 MIGRE Polish Headache Center, Wroclaw, Poland; 16 Department of Neurology, Regional Hospital of Shkodra, Shkoder, Albania; 17 Neurology and Clinical Genetics Department, Kyrgyz State Medical Academy, Bishkek, Kyrgyzstan; 18 General Practice, Moscow, Russia; 19 Neurology Clinic, Department of Neurology, Tartu University Hospital, Tartu, Estonia; 20 Department of Neurology, Third Faculty of Medicine, Charles University and University Hospital Kralovske Vinohrady, Prague, Czech Republic; 21 Department of Neurology, Kingston Hospital, London, UK; 22 Department of Neurology, St George’s University Hospital, London, UK; 23 Department of Neurology, School of Medicine, Istanbul Medipol University, Istanbul, Turkey; 24 Department of Neurology, University Hospital Center Osijek, Osijek, Croatia; 25 Department of Anatomy and Neuroscience, Faculty of Medicine, University J.J. Strossmayer, Osijek, Croatia; 26 Division of Vascular Medicine and Pharmacology, Department of Internal Medicine, Erasmus University Medical Center, Rotterdam, The Netherlands; 27 Department of Neurology, University Emergency Hospital of Bucharest, Bucharest, Romania; 28 Department of Neurosciences, Università Cattolica del Sacro Cuore, Rome, Italy; 29 Neurofisiopatologia, Dipartimento Di Scienze Dell’invecchiamento, Neurologiche, Ortopediche E Della Testa-Collo, Fondazione Policlinico Universitario Agostino Gemelli IRCCS, Rome, Italy; 30 Headache Center, Department of Advanced Medical and Surgical Sciences, University of Campania “Luigi Vanvitelli”, Naples, Italy; 31 Department of Neurology, University Medicine Greifswald, Greifswald, Germany; 32 Neuroscience Section, Department of Applied Clinical Sciences and Biotechnology, University of L’Aquila, L’Aquila, Italy; 33 Departement of Neurology, Headache-Center, Charité - Universitaetsmedizin Berlin, Berlin, Germany; 34 Headache Center, Educational and Scientific Medical Complex, University Clinic” of Karkiv, National Medical University, Karkiv, Ukraine; 35 Neurology and Neurophysiology Service - Pain Medicine Unit, Santa Maria Maddalena Hospital, Occhiobello, Italy; 36 Department of Psychiatry, Division of Consultation Psychiatry and Neuroscienc, Wroclaw Medical University, Wroclaw, Poland; 37 Department of Clinical and Molecular Medicine, Sapienza University, Rome, Italy; 38 Regional Referral Headache Center, Sant’Andrea University Hospital, Rome, Italy; 39 UOC Neurologia, Salute Pubblica, Disabilità, Fondazione IRCCS Istituto Neurologico Carlo Besta, Milan, Italy

**Keywords:** SARS-CoV-2, COVID-19, Vaccination, Headache, BNT162b2, ChAdOx1, Headache, Adverse Event

## Abstract

**Background:**

Vaccines against severe acute respiratory syndrome coronavirus 2 (SARS-CoV-2) are used to reduce the risk of developing Coronavirus Disease 2019 (COVID-19). Despite the significant benefits in terms of reduced risk of hospitalization and death, different adverse events may present after vaccination: among them, headache is one of the most common, but nowadays there is no summary presentation of its incidence and no description of its main features.

**Methods:**

We searched PubMed and EMBASE covering the period between January 1^st^ 2020 and August 6^th^, 2021, looking for record in English and with an abstract and using three main search terms (with specific variations): COVID-19/SARS-CoV-2; Vaccination; headache/adverse events. We selected manuscript including information on subjects developing headache after injection, and such information had to be derived from a structured form (i.e. no free reporting). Pooled estimates and 95% confidence intervals were calculated. Analyses were carried out by vaccine vs. placebo, by first vs. second dose, and by mRNA-based vs. “traditional” vaccines; finally, we addressed the impact of age and gender on post-vaccine headache onset.

**Results:**

Out of 9338 records, 84 papers were included in the review, accounting for 1.57 million participants, 94% of whom received BNT162b2 or ChAdOx1. Headache was generally the third most common AE: it was detected in 22% (95% CI 18–27%) of subjects after the first dose of vaccine and in 29% (95% CI 23–35%) after the second, with an extreme heterogeneity. Those receiving placebo reported headache in 10–12% of cases. No differences were detected across different vaccines or by mRNA-based vs. “traditional” ones. None of the studies reported information on headache features. A lower prevalence of headache after the first injection of BNT162b2 among older participants was shown.

**Conclusions:**

Our results show that vaccines are associated to a two-fold risk of developing headache within 7 days from injection, and the lack of difference between vaccine types enable to hypothesize that headache is secondary to systemic immunological reaction than to a vaccine-type specific reaction. Some descriptions report onset within the first 24 h and that in around one-third of the cases, headache has migraine-like features with pulsating quality, phono and photophobia; in 40–60% of the cases aggravation with activity is observed. The majority of patients used some medication to treat headache, the one perceived as the most effective being acetylsalicylic acid.

**Supplementary Information:**

The online version contains supplementary material available at 10.1186/s10194-022-01400-4.

## Introduction

In late 2019, SARS-CoV-2 (Severe Acute Respiratory Syndrome Coronavirus 2), the etiologic agent of Coronavirus Disease 2019 (COVID-19) spread rapidly into the human population causing a global pandemic, with a relevant impact on mortality but also on a multi-organ morbidity [1]. Despite the effort to control the disease through effective testing and prevention measures like isolation, quarantine, and clinical care of affected individuals, infections continued at an unabated pace resulting in millions of deaths [2] suggesting that effective vaccines are needed to bring the pandemic under control. COVID-19 vaccines are intended to provide acquired immunity preventing symptomatic illness caused by SARS-CoV-2. The already established knowledge about the structure and function of coronaviruses causing diseases like Severe Acute Respiratory Syndrome (SARS) and Middle East respiratory syndrome (MERS) accelerated the development of various vaccine platforms during early 2020 [3] and COVID-19 vaccination became a governmental priority for many countries.

According to recent data published by the U.S Centre for Disease Control, referred to 13 jurisdictions and to the period April 4 – July 17 2021, 92% of infections, 92% of hospitalizations and 91% of deaths were observed among not fully vaccinated (i.e. one dose of two-doses products) or unvaccinated people compared to fully vaccinated (i.e. two doses or one dose of one-shot products) [4] proving that vaccination is an effective way to combat the pandemic. Even when, during 2021, the Delta variant became dominant, the vaccines still protected against severe illness and hospitalizations, although with slight reduction in effectiveness as compared to the original virus [5]. Data published by the COVID-19 Associated Hospitalization Surveillance Network (COVID-NET) show that unvaccinated subjects are 17 times more likely to be hospitalized, even when the Delta variant became predominant: thus vaccines keep on playing a critical role in the prevention of hospitalization and serious complications related to SARS-CoV-2 infection [6].

The first vaccines against COVID-19 were introduced in some countries in the second half of 2020 [7]. Since then at least 22 different preparations have been entered into use, while over a hundred have been submitted for clinical trial databases. Vaccinations accepted or currently considered by the World Health Organisation for emergency use listing are shown in Table 1 [8]. These preparations use very different ways to achieve immunogenicity. The more traditional vaccines are based on Vero cells lines for SARS-CoV-2 replication, i.e. similar to polio or rabies vaccines. However, effective COVID-19 prevention has called for formerly less widely used tools, including virus vectors or messenger ribonucleic acid (mRNA). The combination of novelty, necessary pace and scale of vaccine roll-out raised questions regarding safety. However, after billions of doses administered, it has been confirmed that these fears were mostly unfounded. The vast majority of side effects are mild and include fever, fatigue, headache, muscle pain, chills, diarrhoea and pain at the injection site, and usually disappear in few days after the vaccination. Serious adverse events related to vaccinations are extremely rare. They may include anaphylaxis [9], myocarditis and pericarditis [10] (after mRNA vaccines) or provoke thrombosis with thrombocytopenia syndrome [11] (after adenovirus vector-based vaccines), and possibly Guillain-Barré syndrome [12].

**Table 1:** Covid-19 vaccines submitted for WHO emergency use listing

Name	Company	Date of WHO Emergency Use Listing	Platform
Comirnaty / Tozinameran / BNT162b2	Pfizer/BioNtech	31 December 2020	Nucleoside modified mRNA
Vaxzevria / Covishield / ChAdOx1 / AZD1222	AstraZeneca / Oxford / Serum Institute of India	16 February 2021	Recombinant ChAdOx1 adenoviral vector encoding the SARS-CoV-2 Spike protein antigen
Ad26.COV 2.S	Janssen-Cilag International	12 March 2021	Recombinant, replication incompetent adenovirus type 26 vector encoding the SARS-CoV-2 Spike protein
Spikevax / mRNA-1273	Moderna Biotech	30 April 2021	mRNA-based vaccine encapsulated in lipid nanoparticle
Sinopharm BIBP COVID-19 vaccine	Sinopharm / Beijing Institute of Biological Products	7 May 2021	Inactivated SARS-CoV-2 produced in Vero cells
CoronaVac / PiCoVacc	Sinovac Life Sciences	1 June 2021	Inactivated SARS-CoV-2 produced in Vero cells
Sputnik V / Gam-COVID-Vac	Gamaleya Research Institute of Epidemiology and Microbiology	pending	Human Adenovirus Vector-based Covid-19 vaccine
Covaxin / BBV152	Bharat Biotech	pending	Whole-Virion Inactivated Vero Cell
Convidecia / AD5-nCOV	CanSino Biologics	pending	Recombinant adenovirus type 5 vector vaccine
Covovax / NVX-CoV2373	Novavax	pending	Recombinant nanoparticle prefusion spike protein formulated with Matrix-M™ adjuvant
Vidprevtyn / VAT00002 / VAT00008	Sanofi Pasteur / GSK	pending	Recombinant baculovirus vector encoding the SARS-CoV-2 Spike protein, adjuvanted
SCB-2019	Clover Biopharmaceuticals	pending	Recombinant SARS-CoV-2 Spike-Trimer fusion protein
CureVac	CureVac / Coalition for Epidemic Preparedness Innovations	pending	mRNA-based vaccine encapsulated in lipid nanoparticle

Headache is one of the most frequently reported adverse events (AEs) after vaccination, with some differences in term of incidence related to different vaccine type and dose. In some recent meta-analyses [13, 14], data from clinical trials on either viral protein subunit, mRNA-based and viral vector vaccines, injection-site pain, headaches and fatigue were the most frequently reported ones, with rates being higher after the second doses. In a cross-sectional study, headache incidence was shown to be higher among recipients with a history of headache compared to those with no history of headache [15].

We will probably have to deal with COVID-19 prevention treatments for the next years, and the definition and incidence of possible side effects is crucial. In addition to being a common post-vaccination AEs, headache constitutes the cardinal symptom of primary headache disorders, which are among the most prevalent and disabling conditions [16], and one of the most underdiagnosed conditions [17, 18]. Therefore, understanding the rates of headaches’ incidence after vaccination against COVID-19 is of relevance to enhance clinician’s knowledge on this AE of COVID vaccines: in fact, there is a lack of synthesis either on its incidence, but also its features, e.g. whether post-vaccine headache has the features of tension-type headache (TTH) or of migraine. The primary aim of this systematic literature review with meta-analysis is therefore to assess the pooled incidence of post-vaccine headache (both after first and second dose); secondary aims include addressing headache incidence by product and vaccine type (i.e. mRNA-based vs “traditional” ones), exploring post-vaccination headache features and addressing the role of age and gender on post-vaccine headache onset.

## Methods

We conducted a literature review with meta-analysis and reported results according to the ‘Preferred Reporting Items for Systematic Reviews and Meta-Analyses’ (PRISMA) [19].

### Search strategy

Search terms had to combine information three main terms, i.e. COVID-19/SARS-CoV-2 AND Vaccination AND headache/adverse events. For each of three main terms, PubMed and EMBASE were searched using either free text and MeSH and EMTREE terms. The PubMed search was synthetically organized in this way: [COVID-19 (MeSH) OR COVID (free search in ti/abs)] AND [COVID-19 Vaccines (MeSH) OR Vaccin* (free search in ti/abs)] AND [headache (MeSH) OR headache (free search in ti/abs) OR trial OR side effect (free search in ti/abs)]. Similarly, the EMBASE search was synthetically organized in this way: [Coronarvirus OR SARS-CoV2 (emtree) OR COVID (free search in ti/abs)] AND [SARS-CoV2 Vaccine (emtree) OR Vaccin* (free search in ti/abs)] AND [headache (emtree) OR headache (free search in ti/abs) OR trial OR side effect (free search in ti/abs)].

PubMed and EMBASE were searched covering the period between January 1^st^ 2020 and August 6^th^, 2021, looking for record in English and with an abstract. The full search strings are reported in the Supplementary file.

Retrieved references were exported as.csv files and imported to Rayyan QRCI [20] for duplicates checking. The set of records was then exported to MS excel for study selection and data extraction.

### Study selection

Retrieved references were equally and randomly assigned to the authors who screened titles and abstracts for eligibility. Nine authors (AR, AMR, ERM, MCas, MK, MRob, MS, MT and MW-P) performed the double check about titles and abstracts eligibility of 20% randomly selected references.

To be eligible and be evaluated in full texts, records had to be referred to primary research and to report, in titles and abstracts, information on headache incidence following vaccination against SARS-CoV-2. Authors were instructed to select records if they: a) referred to vaccine for SARS-CoV-2, or similar terms; b) were primary research articles; c) mentioned safety profile/adverse events; d) mentioned headache post-vaccination. Conversely, authors were instructed to exclude records if they: a) were published before 2020; b) did not have an abstract; c) were not in English; d) were letters, editorials, conference material, book chapters, case reports, literature reviews or meta-analyses; e) were clearly out of topic (i.e. not presenting safety profile or headache/ adverse events post-vaccination for SARS-CoV-2, or not referring to human subjects, i.e. experimental animal model or in vitro); f) referred to pediatric populations (aged below 12) exclusively.

In this phase, the agreement among the judgements of the raters (inter-rater reliability) was estimated with Krippendorff's alpha coefficient (α) ranging from 0 (totally disagree) to 1 (totally agree). In case of disagreement, the record was retained for full-text evaluation. In case α was below 0.70, a second 20% set of reference was submitted to double check.

Eligible references were equally and randomly assigned to the authors who screened full texts for inclusion. For full texts evaluation, studies were excluded if: a) were not available in full text; b) were not in English or published before 2020; c) did not refer primary research on COVID vaccine trials (i.e. case reports, letters, editorials, conference material, book chapters, literature reviews or meta-analyses); d) were not based on adult humans (i.e. were animal/in vitro model, or focused on patients aged below 12 exclusively); e) did not report data on safety issues and adverse events derived from a systematic evaluation, i.e. we excluded studies in which a case report form (CRF), either self-reported or based on structured interview with a health professional, was not used.

Five authors (PMik, MRom, MT, SS and SV) performed a double check on 50% of the full texts with regard to their eligibility and Krippendorff's α was calculated: the choice for such a high rate is due to the large set of co-authors.

### Data extraction

Data extraction was performed through an ad hoc electronic spreadsheet of Microsoft Excel for Windows. Included studies were equally and randomly assigned to the authors who extracted information on the sample considering the whole sample, the sub-sample receiving placebo (where applicable), the sub-sample receiving each vaccine (for studies reporting information on more than one vaccine type), as well as for all subjects receiving any kind of vaccine.

Extracted information included basic information, such as total number of participants, number of females, and average age, as well as the core information, i.e. the number of subjects who developed headache after vaccine or placebo inoculation. Additional information was: the number of subject developing migraine-like headache; the number of subject developing TTH-like headache; the number of subject with history of any headache; the number of subject with any chronic medical condition; the rank position of headache among reported AEs; the average days between injection and headache onset. With the exclusion of the last variables (which had to be reported directly), if the information was not directly available (e.g. females referred as percentage), it had to be calculated. Finally, authors were asked to refer whether the trial was in Phase II, III or IV, and whether the sample was entirely composed of subjects with specific health conditions (e.g. sample entirely composed of oncological patients, or patients with HIV).

A final control measure was made on extracted data: five authors (AMR, AndM, AR, MCas, MW-P) double checked 100% of extracted data. Such a choice was again made in consideration of the large amount of authors selecting full-texts and extracting data.

### Data analysis

We descriptively summarized data reported to provide an overview of the included studies and samples in the studies, using medians and interquartile ranges (IQR) for raw data. The measure of interest was the proportion of subjects who developed headache among those who received vaccination of any kind, or placebo.

The 95% Confidence Intervals (95%CI) were based on Wilson’s procedure [21]. Due to expected heterogeneity (in terms of sample size, the use of specific vaccines and recruitment procedures), pooled analyses were performed using the random-effects models [22], and the pooled estimates were calculated after Freeman-Tukey Double Arcsine Transformation to stabilize variance [23]. Indeed, random-effects models are recommended if eligible studies are characterized by heterogeneous methodology, and thus it is unreasonable to assume that they share a common effect. The heterogeneity among studies was assessed relying on the χ^2^-test, and significant heterogeneity was defined when P-value was below 0.10. Inconsistency was quantified using the I^2^ statistic [24]: I^2^ below 40% indicates no or not relevant heterogeneity; I^2^ comprised between 30 and 60% indicates moderate heterogeneity: I^2^ comprised between 50 and 90% indicates substantial heterogeneity; I^2^ higher than 75% indicates considerable heterogeneity [25]. MS excel, with MetaXL add-on, was used to perform analyses.

Sub-analyses included: analysis of headache development by single vaccines, for those vaccine type in which there were at least three studies; comparison between vaccine recipients (taken as a whole) and placebo recipients; comparison between first and second doses in terms of headache onset (considering all vaccines together); comparison between newly-developed mRNA-based vaccines and traditional vaccines (inactivated and vector-based vaccines) recipients.

Finally, to address the impact of age and sex on headache development, a meta-regression analysis was performed. Mean participants’ age and percentage of females in each single study were entered as moderators for the effect of vaccination or placebo on headache development. Meta-regression can be performed only if at least 6 effect size estimates with corresponding data for moderators are available: thus, it was run only for a portion of compounds and placebo [26].

## Results

**Fig. 1:**
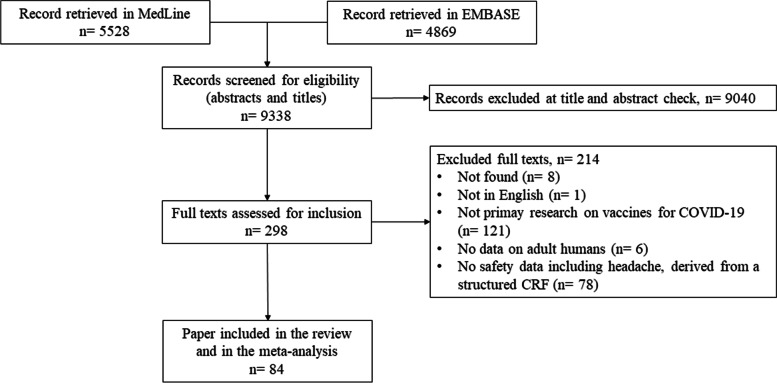
Flowchart of selected studies

Once duplicate were removed, a total of 9338 records were selected for abstract check. Of them, 298 records were retained (double check agreement 98.1%) for full-text evaluation, which resulted in 84 papers included in the review (double check agreement 98.0%) [27–110] (see flowchart in Fig. 1 for paper selection process). Table 2 reports information on the features of different studies. In total, 1.57 million participants were included, most of them were recipients of BNT162b2 [27–51, 76, 78, 79, 81, 83–87, 93, 95, 97, 98, 100, 105, 110] and ChAdOx1 [52–56, 76, 79, 81, 83–87, 92, 93, 97, 98, 100, 105] which together accounted for 94% of the subjects included in the present review. In total 60 out of 84 studies focused on a single vaccine [89–91, 96, 99, 101, 102, 104, 106, 108, 109], 19 on two different vaccines [76–83, 85, 86, 88, 92–95, 97, 100, 105, 110], the remaining on three or more. In terms of sample size, two large population studies included, respectively, 704,003 and 627,383 participants [27, 76], representing alone 85% of all included patients within selected studies. Taken as a whole, a slight female preponderance is reported across the studies. In terms of rank among AEs, headache was generally listed in the third position.

**Table 2:** Features of participants to the different studies: all included subjects, all vaccine recipients, placebo recipients, and single vaccine recipients

	N. of studies	N. of participants	Median female percentage; 25^th^-75^th^ percentile (N. of studies)	Median age; 25^th^-75^th^ percentile (N. of studies)	Median percentage of subjects with chronic conditions; 25^th^-75^th^ percentile (N. of studies)	Median headache rank position among side effects; 25^th^-75^th^ percentile (N. of studies)
All included subjects	84	1,568,199	58.4%; 49.4–69.5% (*N* = 76)	39.8; 35.5–49.9 (*N* = 62)	37.2%; 18.2–92.5% (*N* = 19)	3^rd^; 3^rd^-4^th^ (*N* = 57)
All Vaccines recipients	84	1,522,830	59.4%; 49.1–71.1% (*N* = 72)	41.0; 35.3–48.6 (*N* = 56)	34.7%; 16.9–96.3% (*N* = 22)	3^rd^; 3^rd^-4^th^ (*N* = 56)
BNT162b2	41	1,047,545	64.3%; 52.3–71.7% (*N* = 34)	43.5; 38.2–57.3 (*N* = 26)	85.0%; 39.4–100% (*N* = 9)	3^rd^; 3^rd^-4^th^ (*N* = 32)
ChAdOx1	19	388,147	71.2%; 61.1–76.3% (*N* = 12)	37.5; 35.8–49.7 (*N* = 8)	45.8%; 41.5–50.1% (*N* = 2)	4^th^; 3^rd^-4^th^ (*N* = 12)
PiCoVacc	9	15,177	62.4%; 49.0%-67.7% (*N* = 6)	35.8; 35.4–36.1 (*N* = 5)	15.8%; 15.2–16.3% (*N* = 2)	3^rd^; 2^nd^-3^rd^ (*N* = 7)
mRNA-1273	5	16,575	51.8%; 41.1–64.4% (*N* = 4)	64.7; 48.9–66.7 (*N* = 3)	100%; n.a (*N* = 1)	3^rd^; 2^nd^-3^rd^ (*N* = 3)
NVX-CoV2373	3	7,612	48.8%; 48.7–49.0% (*N* = 2)	56.0; n.a (*N* = 1)	n.r	3^rd^; 2^nd^-3^rd^ (*N* = 2)
Placebo Recipients	21	45,760	52.4%; 47.6–54.2% (*N* = 20)	43.3; 37.6–48.1 (*N* = 19)	13.8%; 4.8–26.9% (*N* = 5)	3^rd^; 2^nd^-4^th^ (*N* = 18)

Vaccines reported by less than three studies were not included in the table

*N.R.* not reported, *N.A.* not available

Table 3 shows a synthesis of the pooled rates of headache onset after injection for SARS-CoV-2 (detailed plots are included in supplementary materials). Data show a higher incidence rate among vaccine recipients compared to placebo recipients, both at the first and at the second dose, with no relevant differences between vaccines type. An exception to this was ChAdOx1 on the first dose, for whom a higher pooled incidence was observed, as well as higher incidence on the first dose compared to the second. In addition, no difference can be appreciated between recipients of the new vaccines, based on mRNA technology (i.e. BNT162b2 and mRNA-1273) contrasted to the “traditional” ones, i.e. those based on inactivated viruses or recombinant viral vectors. Lastly, a relevant heterogeneity across studies was observed for almost all analyses, excluding NVX-CoV2373 at the first dose. In fact, our results tell that 22% (95% CI 18–27%) of subjects developed headache after the first dose of vaccine, the same figure being 29% (95% CI 23–35%) after the second. However, such rates were extremely variable: at the first dose (see supplementary Figure S1), incidence rates varied between 0% (95% CI 0–4%) to 100%, whereas at the second dose (see supplementary Figure S2) rates varied again between 0% (95% CI 0–4%) to 83% (95% CI 65–96%).

**Table 3:** Pooled rates and 95% CI for headache onset after injection against SARS-CoV-2

	N. of studies	Pooled headache incidence (95%CI)	Q (*p*-value)	I^2^
All vaccines recipients, 1^st^ dose	84	22% (18–27%)	270,544.2 (*p* < .01)	100%
All vaccines recipients, 2^nd^ dose	46	29% (23–35%)	16,478.5 (*p* < .01)	100%
Placebo recipients, 1^st^ dose	21	10% (6–13%)	2203.7 (*p* < .01)	99%
Placebo recipients, 2^nd^ dose	10	12% (7–17%)	955.7 (*p* < .01)	99%
mRNA vaccine recipients, 1^st^ dose	43	22% (17–27%)	84,389.3 (*p* < .01)	100%
Traditional vaccine recipients, 1^st^ dose	54	23% (18–29%)	44,406.8 (*p* < .01)	100%
BNT162b2, 1^st^ dose	41	21% (16–27%)	82,671.2 (*p* < .01)	100%
BNT162b2, 2^nd^ dose	26	30% (23–38%)	6784.6 (*p* < .01)	100%
ChAdOx1, 1^st^ dose	19	53% (39–66%)	29,819.7 (*p* < .01)	100%
ChAdOx1, 2^nd^ dose	3	29% (11–51%)	5.4 (*p* = .07)	63%
mRNA-1273, 1^st^ dose	5	28% (10–51%)	64.4 (*p* < .01)	94%
mRNA-1273, 2^nd^ dose	3	54% (32–74%)	12.6 (*p* < .01)	84%
NVX-CoV2373, 1^st^ dose	3	25% (24–26%)	1.5 (*p* = .46)	0%
NVX-CoV2373, 2^nd^ dose	3	31% (18–47%)	80.9 (*p* < .01)	100%
PiCoVacc, 1^st^ dose	9	11% (5–19%)	1283.8 (*p* < .01)	100%

Information on headache onset was generally reported within the first seven days after injection, since this is the timeframe used in most of the trials to collect information on solicited AEs: however, no study reported information on the average days between injection and headache onset. Similarly, none of the studies herein included reported information on headache features, i.e. whether it was TTH-like or migraine-like, as well as its duration or response to acute medications.

Table 4 reports the results of the meta-regression analysis addressing the impact of age and gender on post-vaccination headache. The only retrieved effect shown is a lower prevalence of headache after first injection of BNT162b2 in older participants.

**Table 4:** Results of the Meta-Regression Analysis

	Moderator	k	B	SE	*p*
All vaccines recipients, 1^st^ dose	Age	60	-0.004	0.002	.082
	%females	76	0.048	0.034	.162
Placebo recipients, 1^st^ dose	Age	19	-0.001	0.004	.896
	%females	20	-0.013	0.382	.973
All vaccines recipients, 2^nd^ dose	Age	17	0.001	0.003	.965
	%females	25	0.134	0.189	.485
BNT162b2, 1^st^ dose	Age	26	-0.005	0.002	.041
	%females	33	0.115	0.188	.545
BNT162b2, 2^nd^ dose	Age	10	-0.006	0.004	.213
	%females	15	0.160	0.320	.625
PiCoVacc, 1^st^ dose	%females	6	0.242	0.272	.424
ChAdOx1, 1^st^ dose	Age	8	-0.012	0.009	.231
	%females	12	0.688	0.435	.145

k refers to the number of studies included in specific meta-regression analyses

## Discussion

With this systematic review with meta-analysis we analysed the pooled incidence rates of headache onset after vaccination against SARS-CoV-2, or placebo receipt. Our results show that 22% (95% CI 18–27%) of subjects developed headache after the first dose of vaccine and 29% (95% CI 23–35%) after the second, and that these rates were two-fold higher compared to those receiving placebo (10–12%). Headache was generally reported as the third most common AE, the first usually being pain at injection site. No significant differences could be observed between vaccines based on the new mRNA technology and “traditional” (i.e. inactivated viruses or recombinant viral vectors) ones, together with a minor impact of age, which enables presuming that headache is secondary to systemic immunological reaction than to a vaccine-type specific reaction.

After almost two years since the beginning of the COVID-19 pandemic, the SARS-CoV-2 is still causing a large number of deaths worldwide, and vaccination campaign is the most promising and safe way to reduce its spreading: this in fact means to reduce the impact of COVID-19 on the health of citizens [111], by reducing mortality but also by reducing morbidity and long term impact of COVID-19, and in particular to protect the health and wellbeing of the weakest ones [112]. Like all vaccines, also those used to prevent SARS-CoV-2 spreading and the risks connected to the development of COVID-19 may produce AEs: among them, headache is one of the most common. The fact that it is more associated to vaccine than to placebo indicates an effect of the vaccine itself on headache development. This is also witnessed by the fact that population studies addressing rates of headache in a short-time timeframe (i.e. the so-called “headache yesterday” approach) showed headache prevalence ranging between 6 and 17% [113–116]. Such a kind result, however, deserve some comments.

First, included studies report largely heterogeneous results on onset of post-vaccination headache, with no information about pre-existing headache disorders, which might act as risk factors for post-vaccination headache development. Moreover, no information on headache features, time to onset, duration and response to acute treatment could be derived by included studies. So, our literature review cannot provide direct evidence on pre-existing headache and features of post-vaccination headache, which is fundamental to determine if headache occurring after vaccination against SARS-CoV-2 can be considered as one of the usual headaches (for those with previous history of headache), or a totally new headache.

The main reason for such a poor description of headache features lies in the selection criteria we applied, i.e. that we selected studies focused on vaccine efficacy, in which headache was addressed within solicited AEs, i.e. using structured CRFs. In most cases, such AEs were collected within 7 days from injection: this clearly leaves poor data to present the features of such headache, and thus such data do not enable to distinguish reversible, common and non-severe cases from those that should be considered as red flags for life threatening conditions, like cerebral venous thrombosis (CVT). Some reports specifically address headache as a premonitory symptom of CVT in non-replicant adenovirus vector based COVID-19 vaccine recipients [117, 118]. Such kind of headaches’ features is reported as severe, progressive and treatment-resistant, the main characteristic is being delayed from vaccination (i.e. usually more than a week). So, patients developing a new-onset headache, around a week after vaccination with an adenovirus vector-based vaccine, with or without other neurological symptoms should be carefully followed up for the risk of developing cerebral venous thrombosis: risk factors also include thrombocytopenia, anti-platelet factor 4 antibodies, and multiple organ thrombosis, i.e. vaccine-induced immune thrombotic thrombocytopenia.

However, in most cases, headache occurring after vaccination from SARS-CoV-2 is reversible, with onset few hours after vaccine receipt and a few hours to few days’ duration. Two manuscripts showed differences between first and second dose: our results show headache is more common after the second dose, but one study by Perrotta and colleagues showed the opposite (29.2% after the first and 22.1% after the second) [119]. The seven days’ period is a standard for AEs detection in clinical studies addressing vaccine efficacy or in studies addressing safety profile. However, in such a period a patient with pre-existing headache disorder, especially those with high frequency or chronic ones, is likely to have a headache episode irrespectively of vaccination status. Therefore, these patients might have difficulties to differentiate between post-vaccination headaches and their usual episodes when just responding to a structured CRF which is not intended to address such a difference. Nevertheless, two studies (not included in our review) showed higher rates of headache onset among patients with a history of headache disorders: Sekiguchi and colleagues reported that 13.6% and 32% of those without headache history, and 30.9% and 66.2% of those with headache history developed post-vaccination headache after the first and second dose, respectively [15]; Ekizoglu and colleagues reported that 21.1% of those without headache history, and 38.8% of those with headache history developed post-vaccination headache [120].

Finally, with regard to the features of headache occurring after SARS-CoV-2 vaccines, some inputs were available in four different studies [15, 53, 120, 121], and the main characteristics were described in Table 5. In synthesis, such headaches generally onset within the first day from injection and its duration is below 24 h. It is bilateral in 70–75% of the cases, and pulsating quality is reported by 30–40% of patients. Aggravation with activity is the most common accompanying symptom, followed by phono/photophobia, nausea and, in less than 5% of reported cases, by osmophobia; headache intensity is generally a moderate one. Taken as a whole, the features of such a headache do not resemble migraine-like one in most of the cases: however, but in approximately one third of the cases, migrainous features seem to be met, and this seems to be much more common among people with pre-existing migraine. The headache incidence increase shown in our study thus supports the need to make an effort for classifying headache arising after vaccination against SARS-CoV-2, so as to differentiate these headaches from other spontaneously occurring headache episodes, and to identifying those that might constitute a red flag for CVT.

**Table 5:** Main features of post-vaccine headache described in available literature

	Sekiguchi [15]^a^	Ekizoglu [113]	Göbel [121]	Göbel [53]
Subjects developing headache after vaccination against SARS-CoV-2	78	556	2349	2464
Females	n.r	441 (79%)	1289 (74%)	1534 (85%)
Age (mean ± SD)	n.r	43.4 ± 12.3	41.0 ± 11.6	39.0 ± 12.7
Accompanying symptoms				
Aggravation with activity	49 (63%)	137 (25%)	1010 (43%)	1232 (50%)
Phonophobia	16 (21%)	83 (15%)	658 (28%)	813 (33%)
Photophobia	6 (8%)	94 (17%)	634 (27%)	788 (32%)
Nausea	14 (18%)	67 (12%)	564 (24%)	690 (28%)
Osmophobia	n.r	22 (4%)	70 (3%)	99 (4%)
Headache severity				
Mild		126 (23%)	328 (14%)	267 (11%)
Moderate		370 (66%)	1081 (46%)	867 (35%)
Severe		60 (11%)	940 (40%)	1330 (54%)
NRS^b^	5 (IQR: 4–8)			
Headache features				
Unilateral location	20 (26%)	184 (33%)	634 (27%)	591 (24%)
Pulsating quality	39 (50%)	223 (40%)	681 (29%)	838 (34%)
Time to onset (hours; mean ± SD)	12 (IQR: 7–18)	43 ± 84	18 ± 27	15 ± 22
Duration (hours; mean ± SD)	8 (IQR: 4–24)	12 (IQR: 5–72)	14 ± 21	16 ± 30
Need for drug use	68 (87%)	385 (70%)	1396 (59%)	1960 (80%)

^a^information was related to the second dose; ^b^NRS on a 0–10 scale. Categorical variables are reported as frequencies and percentages; continuous variables as means ± standard deviation (unless differently stated). None of the studies from the present table was included in the literature review with meta-analysis

With regard to treatment for post-vaccination headache, available literature provides little information. The aforementioned studies [15, 53, 120, 121] show that the majority of patients had used analgesics to treat it, but distinction between patients with and without pre-existing headache is not reported. Partial or complete response was reported by 97% of patients who took analgesics in the study of Ekizoglu [120], whereas in the two studies of Göbel and colleagues [53, 121] it was shown that the two most commonly used drugs were paracetamol and ibuprofen (respectively used by 45.5% and 36% of subjects), whereas the one perceived being the most effective, by 46.2% of users, was acetylsalicylic acid. There is therefore pending issues about the proposed treatments as over the counter medications, that usually alleviate also fever, may exert a partial effect on patients with a history of migraine.

The possible mechanism of post-vaccination headache is currently unknown and, in order to make some hypotheses, a step back to the mechanisms that have been hypothesized for SARS-CoV-2 to spread and impact on human body is needed. Several possible routes for SARS-CoV-2 virus spread in the human body are currently postulated: use of the bloodstream with subsequent neuronal dissemination, infection of endothelial cells within the blood–brain barrier (BBB) or blood-cerebrospinal fluid barrier, use of transsynaptic pathways after infection of the endings nerves (forward or retrograde transport) mainly in the olfactory bulb, crossing the BBB as a result of leukocyte infection (Trojan horse mechanism) or through the use of the glymphatic system [122, 123]. However, it seems that the most likely receptor mechanism is the use of angiotensin-converting enzyme type 2 (ACE2) to break BBB. ACE2 expression outside the lung tissue was confirmed in neurons, astrocytes, oligodendrocytes, olfactory bulb, substantia nigra, brainstem, posterior cingulate cortex, striatum, and hypothalamus [124].

SARS-CoV-2 has been shown to be a neurotropic virus that has the ability to infect and replicate in cultures of neuronal cells and brains [125]. It seems that the emergence of neurological symptoms in COVID-19 occurs through three possible mechanisms: direct viral invasion, immune-mediated post-inflammatory complications, and a mechanism secondary to lung damage and systemic disease [126]. Although the main symptoms of COVID-19 are fever, cough and shortness of breath, one of the most frequently observed symptoms preceding or occurring during and after SARS-CoV2 infection is headache [127]. Indeed, in COVID-19, headache is the most frequently reported neurological symptom, with a prevalence of 10–37% [128, 129] in studies based in hospital setting. However, headache seems to be much more prevalent (39–72%) when analysed in prospective studies by structured questionnaires [130–133]. The mechanism by which headache occurs in COVID-19 remains unclear. Some authors suggest that the headache may be a consequence of direct activation of the trigemino-vascular system by the SARS-CoV2 or through the increased circulating proinflammatory cytokines and hypoxia or vasculopathy [134]. Primary headache associated with COVID-19 infection can be a consequence of increased stress related to infection or post-traumatic stress disorder related to COVID-19, which may be a trigger for headache or de novo headache related to infection (new daily persistent headache). Secondary headache can be associated with cytokine release syndrome, systemic viral infection and other causes related to direct COVID-19 infection either vascular or non-vascular [135].

COVID-19 related headache is frequently accompanied by osmophobia, phonophobia, and photophobia, which are less frequently reported in post-vaccination headache. In addition, other clinical features may present with some differences: post-vaccination headache is in fact mostly bilateral, less intensity and shorter duration than COVID-19 related headache [120]. Post-vaccination headache could be secondary to substances administered during immunisation. However, this seems unlikely considering the diversity of vaccine types included in our study, and due to the general lack of age and gender effect on headache onset. Inactivated virus vaccines differ in composition from vector-based. These in turn are incomparable to mRNA-based agents. This difference in composition should hypothetically translate to differences in headache prevalence and phenotype after separate vaccine types. However, our study did not find significant differences in headache prevalence after miscellaneous vaccination types.

Probably the only common denominator for all of the analysed vaccine types is SARS-CoV-2 spike protein (SP). SP binds with ACE2 and leads to diverse biological, and potentially pain-related reactions [134]. Presently, it is unknown whether ACE2 is present in peripheral structures of the trigeminal nerve, although some other neuronal structures express ACE2 [127, 136]. If indeed SP was causing headache, then after vaccination with an inactivated virus this symptom should have an earlier onset than after other agents, as mRNA and vector based vaccines require a transcription phase for the spike protein to occur in the system. Unfortunately, the studies included in our meta-analysis do not provide data on an exact headache timeline. However, some large retrospective observations suggest that headache starts several hours after vaccination and remits in most cases within few hours [15, 53, 120, 121]. It should be underlined that the results of our meta-analysis to some extent exclude SP as the cause of headache. This can be deduced from the fact that headache was more prevalent after the second vaccine dose. If SP was the cause of headache, then neutralising antibodies present after the first dose should decrease levels of circulating SP after the second dose, and consequently diminish the prevalence of headache.

There exists an alternative to above described rationale. It attributes this symptom to disorder of homoeostasis. Vaccines are designed to induce humoral and cellular immunity. These reactions include diverse mechanisms, some of which possibly may provoke headache. Adaptive immune reaction takes days or weeks to develop, and hence would not provoke headache occurring hours after vaccination. However, innate immunity has been proven to begin within hours after vaccine administration [137, 138]. This type of reaction includes many cytokines mediating an acute-phase reaction. Serum concentrations of some cytokines (interferon gamma, interleukin-6, C-X-C motif chemokine ligand 10) have been shown to peak early after vaccination and decrease within several days [138]. Moreover, these cytokines were shown by the same study to achieve even higher concentrations after the second vaccine dose. This could explain a higher prevalence of headache after the second immunisation. Furthermore, other symptoms related to innate immune reaction (i.e. fever and myalgia) have been associated with a (mostly) virus-based vaccine [120]. Finally, the mechanism associated with innate immune reaction explains to some extent the similarities in headache prevalence after vaccines against other viruses, i.e. influenza [139].

The attribution of headache to immune reaction is a compelling idea. It could be hypothesised that in this situation a more robust immune reaction could translate to more headache cases, but also more effective immunogenicity. Alas the studies from our meta-analysis did not try to find an association between post-vaccination headache and vaccine effectiveness. A post-hoc analysis could solve this conundrum. Finally, it should be mentioned that both of above-described mechanisms (SP and innate immune reaction) could explain many similarities between headache characteristics after COVID-19 vaccination [15, 53, 120, 121] and after COVID-19 itself [120, 133, 140].

When discussing the possible mechanism of post-vaccination headache, one might wonder if, in the case of viral vector COVID-19 vaccines, the headache is not a consequence of systemic infection. Then the headache could be identified using ICHD-3 code 9.9.2.1 – Acute headache attributed to systemic viral infection. However, in such a case, one would expect a higher incidence of headaches from this type of vaccination, which was not observed in this meta-analysis. That is naturally a completely theoretical situation, since adenoviruses in vector-based vaccines are unable to replicate. Hence their spread is limited to the muscle where they were administered via injection.

It should also be remembered that in very rare cases the headache following vaccination may be a consequence of a complication of the COVID-19 vaccination. Cases of cerebral venous thrombosis or ischemic stroke in unusual locations associated with vaccine-induced immune thrombotic thrombocytopenia (VITT) have been reported following ChAdOx1-S and Ad26.COV2S vaccine [141, 142]. The pathophysiology of VITT is presumably the development of immunoglobulin G antibodies against platelet factor 4, further resulting in platelet consumption and thrombus formation. Symptoms of thrombosis, thrombocytopenia or coagulation abnormalities appear within five to ten days post-vaccination and are a late complication of vaccination [142]. Consequently, any headache secondary to VITT should occur or recur 5–10 days after immunisation.

Some limitations have to be taken into consideration. First, we were unable to locate eight studies. Second, there was a considerable heterogeneity across studies and two very large studies account for approximately 85% of the studies’ sample (however, in terms of weights the contribution is well balanced and they did not account for excess weight). Third, there is a mixture of phase-II/III and phase-IV studies, and therefore comparison against placebo is not always reported. Fourth, we were unable to report information on headache type and duration, and on presence of headache before vaccination, as this kind of information was not reported among selected studies: the information reported in this manuscript referred to headache features and higher likelihood to develop it among patients with history of headache was in fact derived from descriptive studies which did not meet our inclusion criteria. Finally, we have no information at all whether being on a migraine-specific prophylaxis might have a positive effect on post-vaccine headache development.

## Conclusions

In conclusion, headache is the third most common AE associated to vaccination against SARS-CoV-2, and it occurred in 22% (95% CI: 18–27%) after the first and in 29% (95% CI: 23–35%) after the second dose over a 7-day period, and such rates are higher compared to those receiving placebo (10–12%) as well as compared to population studies showing 6–17% likelihood to have headache on the previous day. The features of such headache, in approximately one-third of the cases, resemble that of migraine with pulsating quality, phono and photophobia, whereas aggravation with activity is more common (generally in 40–60% of the cases). Its onset is generally within the first 24 h and the majority of patients used some medication to treat headache, the one perceived as the most effective being acetylsalicylic acid.

Our meta-analysis showed no significant difference in the frequency of headaches observed with different vaccine types, which differ essentially in composition. Given the various mechanisms possibly implicated in the onset of post-vaccination headache, it seems most likely to be related to the systemic inflammatory response with a still unclear mechanism.

## Supplementary Information

**Additional file 1.**

## Data Availability

Not applicable.

## Abbreviations

ACE2: Angiotensin-Converting Enzyme Type 2
AE: Adverse Event
BBB: Blood–Brain Barrier
COVID-19: Coronavirus Disease 2019
CRF: Case Report Form
CVT: Cerebral Venous Thrombosis
IQR: Interquartile Range
MERS: Middle East respiratory syndrome
mRNA: Messenger Ribonucleic Acid
PRISMA: Preferred Reporting Items for Systematic Reviews and Meta-Analyses
SARS: Severe Acute Respiratory Syndrome
SARS-CoV-2: Severe Acute Respiratory Syndrome Coronavirus 2
SP: Spike Protein
TTH: Tension-Type Headache
VITT: Vaccine-Induced Immune Thrombotic Thrombocytopenia
95%CI: 95% Confidence Intervals

## References

[1] Kaur N, Gupta I, Singh H (2020). Epidemiological and clinical characteristics of 6635 covid-19 patients: a pooled analysis. SN Compr Clin Med.

[2] WHO coronavirus disease (COVID-19) dashboard. https://covid19.who.int/table

[3] Li Y-D, Chi W-Y, Su J-H (2020). Coronavirus vaccine development: from SARS and MERS to COVID-19. J Biomed Sci.

[4] Scobie HM, Johnson AG, Suthar AB (2021). Monitoring incidence of COVID-19 cases, hospitalizations, and deaths, by vaccination status — 13 U.S. jurisdictions, April 4–July 17, 2021. MMWR Morb Mortal Wkly Rep.

[5] Tartof SY, Slezak JM, Fischer H (2021). Effectiveness of mRNA BNT162b2 COVID-19 vaccine up to 6 months in a large integrated health system in the USA: a retrospective cohort study. Lancet E-Pub.

[6] Havers FP, Pham H, Taylor CA, et al. COVID-19–associated hospitalizations among vaccinated and unvaccinated adults ≥18 years—COVID-NET, 13 states, January 1–July 24, 2021. medRxiv [Preprint posted online August 29, 2021]

[7] Balakrishnan VS (2020). The arrival of Sputnik V. Lancet Infect Dis.

[8] Status of COVID-19 Vaccines within WHO EUL/PQ evaluation process. https://extranet.who.int/pqweb/sites/default/files/documents/Status_COVID_VAX_19August2021.pdf

[9] Phillips EJ (2021). Allergic reactions after COVID-19 vaccination—putting risk into perspective. JAMA Netw Open.

[10] Diaz GA, Parsons GT, Gering SK (2021). Myocarditis and pericarditis after vaccination for COVID-19. JAMA.

[11] Franchini M, Liumbruno GM, Pezzo M (2021). COVID-19 vaccine-associated immune thrombosis and thrombocytopenia (VITT): diagnostic and therapeutic recommendations for a new syndrome. Eur J Haematol.

[12] Dyer O (2021). Covid-19: regulators warn that rare Guillain-Barré cases may link to J&J and AstraZeneca vaccines. BMJ.

[13] Cai C, Peng Y, Shen E (2021). A comprehensive analysis of the efficacy and safety of COVID-19 vaccines. Mol Ther.

[14] Kadali RAK, Janagama R, Peruru S (2021). Non-life-threatening adverse effects with COVID-19 mRNA-1273 vaccine: a randomized, cross-sectional study on healthcare workers with detailed self-reported symptoms. J Med Virol.

[15] Sekiguchi K, Watanabe N, Miyazaki N (2021). Incidence of headache after COVID-19 vaccination in patients with history of headache: a cross-sectional study. Cephalalgia E-Pub.

[16] Steiner TJ, Stovner LJ, Jensen R (2020). Migraine remains second among the world’s causes of disability, and first among young women: findings from GBD2019. J Headache Pain.

[17] Arroyo-Quiroz C, Kurth T, Cantu-Brito C (2014). Lifetime prevalence and underdiagnosis of migraine in a population sample of Mexican women. Cephalalgia.

[18] Fermo OP (2021). Underdiagnosis and undertreatment of migraine in Poland. Neurol Neurochir Pol.

[19] Moher D, Liberati A, Tetzlaff J, Altman DG, PRISMA Group (2009). Preferred reporting items for systematic reviews and meta-analyses: the PRISMA statement. Ann Intern Med.

[20] Ouzzani M, Hammady H, Fedorowicz Z, Elmagarmid A (2016). Rayyan—a web and mobile app for systematic reviews. Syst Rev.

[21] Newcombe RG (1998) Two-sided confidence intervals for the single proportion: comparison of seven methods. Stat Med 17:857–872. 10.1002/(sici)1097-0258(19980430)17:8%3c857::aid-sim777%3e3.0.co;2-e 10.1002/(sici)1097-0258(19980430)17:8<857::aid-sim777>3.0.co;2-e 9595616

[22] DerSimonian R, Laird N (1986). Meta-analysis in clinical trials. Control Clin Trials.

[23] Freeman MF, Tukey JW (1950). Transformations related to the angular and the square root. Ann Math Stats.

[24] Higgins JP, Thompson SG, Deeks JJ, Altman DG (2003). Measuring inconsistency in meta-analyses. BMJ.

[25] Higgins JPT, Green S (editors). Cochrane Handbook for Systematic Reviews of Interventions version 5.1.0. Cochrane, 2011. Available from https://handbook-5-1.cochrane.org/front_page.htm, last access 15/10/2021

[26] Fu R, Gartlehner G, Grant M, et al. (2010) Conducting Quantitative Synthesis When Comparing Medical Interventions: AHRQ and the Effective Health Care Program. In: Agency for Healthcare Research and Quality. Methods Guide for Comparative Effectiveness Reviews. Rockville, MD. Available at: http:​//effectivehealthcare.ahrq.gov/ (last access 28/01/2022) 21433407

[27] García-Grimshaw M, Ceballos-Liceaga SE, Hernández-Vanegas LE (2021). Neurologic adverse events among 704,003 first-dose recipients of the BNT162b2 mRNA COVID-19 vaccine in Mexico: a nationwide descriptive study. Clin Immunol.

[28] Polack FP, Thomas SJ, Kitchin N (2020). Safety and efficacy of the BNT162b2 mRNA Covid-19 vaccine. N Engl J Med.

[29] Frenck RW, Klein NP, Kitchin N (2021). Safety, immunogenicity, and efficacy of the BNT162b2 Covid-19 vaccine in adolescents. N Engl J Med.

[30] d’Arminio Monforte A, Tavelli A, Perrone PM (2021). Association between previous infection with SARS CoV-2 and the risk of self-reported symptoms after mRNA BNT162b2 vaccination: data from 3,078 health care workers. EClinicalMedicine.

[31] Baldolli A, Michon J, Appia F (2021). Tolerance of BNT162b2 mRNA COVI-19 vaccine in patients with a medical history of COVID-19 disease: a case control study. Vaccine.

[32] Ossato A, Tessari R, Trabucchi C (2021). Comparison of medium-term adverse reactions induced by the first and second dose of mRNA BNT162b2 (Comirnaty, Pfizer-BioNTech) vaccine: a post-marketing Italian study conducted between 1 January and 28 February 2021. Eur J Hosp Pharm.

[33] Nittner-Marszalska M, Rosiek-Biegus M, Kopeć A (2021). Pfizer-BioNTech COVID-19 vaccine tolerance in allergic versus non-allergic individuals. Vaccines (Basel).

[34] Cuschieri S, Borg M, Agius S (2021). Adverse reactions to Pfizer-BioNTech vaccination of healthcare workers at Malta’s state hospital. Int J Clin Pract.

[35] Izumo T, Kuse N, Awano N (2021). Side effects and antibody titer transition of the BNT162b2 messenger ribonucleic acid coronavirus disease 2019 vaccine in Japan. Respir Investig.

[36] Riad A, Pokorná A, Attia S (2021). Prevalence of COVID-19 vaccine side effects among healthcare workers in the Czech Republic. J Clin Med.

[37] Furer V, Eviatar T, Zisman D (2021). Immunogenicity and safety of the BNT162b2 mRNA COVID-19 vaccine in adult patients with autoimmune inflammatory rheumatic diseases and in the general population: a multicentre study. Ann Rheum Dis.

[38] Kadali RAK, Janagama R, Peruru S (2021). Side effects of BNT162b2 mRNA COVID-19 vaccine: a randomized, cross-sectional study with detailed self-reported symptoms from healthcare workers. Int J Infect Dis.

[39] Borobia AM, Carcas AJ, Pérez-Olmeda M (2021). Immunogenicity and reactogenicity of BNT162b2 booster in ChAdOx1-S-primed participants (CombiVacS): a multicentre, open-label, randomised, controlled, phase 2 trial. Lancet.

[40] Bookstein Peretz S, Regev N, Novick L (2021). Short-term outcome of pregnant women vaccinated with BNT162b2 mRNA COVID-19 vaccine. Ultrasound Obstet Gynecol.

[41] El-Shitany NA, Harakeh S, Badr-Eldin SM (2021). Minor to moderate side effects of Pfizer-BioNTech COVID-19 vaccine among Saudi residents: a retrospective cross-sectional study. Int J Gen Med.

[42] Morales-Núñez JJ, Muñoz-Valle JF, Meza-López C (2021). Neutralizing Antibodies Titers and Side Effects in Response to BNT162b2 Vaccine in Healthcare Workers with and without Prior SARS-CoV-2 Infection. Vaccines (Basel).

[43] Lee YW, Lim SY, Lee JH (2021). Adverse reactions of the second dose of the BNT162b2 mRNA COVID-19 vaccine in healthcare workers in Korea. J Korean Med Sci.

[44] Lotan I, Wilf-Yarkoni A, Friedman Y (2021). Safety of the BNT162b2 COVID-19 vaccine in multiple sclerosis (MS): early experience from a tertiary MS center in Israel. Eur J Neurol.

[45] Simon B, Rubey H, Treipl A (2021). Haemodialysis patients show a highly diminished antibody response after COVID-19 mRNA vaccination compared with healthy controls. Nephrol Dial Transplant.

[46] Pimpinelli F, Marchesi F, Piaggio G (2021). Fifth-week immunogenicity and safety of anti-SARS-CoV-2 BNT162b2 vaccine in patients with multiple myeloma and myeloproliferative malignancies on active treatment: preliminary data from a single institution. J Hematol Oncol.

[47] Cserep G, Morrow D, Latchford K (2021). The effect of a single dose of BNT162b2 vaccine on the incidence of severe COVID-19 infection in patients on chronic hemodialysis: a single-centre study. Clin Exp Nephrol.

[48] Abohelwa M, Elmassry M, Abdelmalek J et al (2021) 2019 Novel coronavirus vaccination among post-graduate residents and fellows. J Prim Care Community Health 12:21501327211022976. 10.1177/21501327211022978 10.1177/21501327211022978 PMC8170335 34053350

[49] Peled Y, Ram E, Lavee J (2021). BNT162b2 vaccination in heart transplant recipients: clinical experience and antibody response. J Heart Lung Transplant.

[50] Sahin U, Muik A, Derhovanessian E (2020). COVID-19 vaccine BNT162b1 elicits human antibody and TH1 T cell responses. Nature.

[51] Achiron A, Dolev M, Menascu S (2021). COVID-19 vaccination in patients with multiple sclerosis: what we have learnt by February 2021. Mult Scler.

[52] Tobaiqy M, Elkout H, MacLure K (2021). Analysis of thrombotic adverse reactions of COVID-19 AstraZeneca vaccine reported to EudraVigilance database. Vaccines (Basel).

[53] Göbel CH, Heinze A, Karstedt S (2021). Headache attributed to vaccination against COVID-19 (Coronavirus SARS-CoV-2) with the ChAdOx1 nCoV-19 (AZD1222) vaccine: a multicenter observational cohort study. Pain Ther.

[54] Jeon M, Kim J, Oh CE (2021). Adverse events following immunization associated with coronavirus disease 2019 vaccination reported in the mobile vaccine adverse events reporting system. J Korean Med Sci.

[55] Pokharel K, Dawadi BR, Karki A (2021). Side effects after second dose of covishield vaccine among health care workers: a descriptive cross sectional study. JNMA J Nepal Med Assoc.

[56] Logunov DY, Dolzhikova IV, Zubkova OV (2020). Safety and immunogenicity of an rAd26 and rAd5 vector-based heterologous prime-boost COVID-19 vaccine in two formulations: two open, non-randomised phase 1/2 studies from Russia. Lancet.

[57] Tanriover MD, Doğanay HL, Akova M (2021). Efficacy and safety of an inactivated whole-virion SARS-CoV-2 vaccine (CoronaVac): interim results of a double-blind, randomised, placebo-controlled, phase 3 trial in Turkey. Lancet.

[58] Wang G, Zhu L, Zhu Y (2021). Safety survey by clinical pharmacists on COVID-19 vaccination from a single center in China. Hum Vaccin Immunother.

[59] Avcı H, Karabulut B, Eken HD (2021). Otolaryngology-specific symptoms may be highly observed in patients with a history of Covid-19 infection after inactivated coronavirus vaccination. Ear Nose Throat J.

[60] Zhang MX, Zhang TT, Shi GF (2021). Safety of an inactivated SARS-CoV-2 vaccine among healthcare workers in China. Expert Rev Vaccines.

[61] Djanas D, Yusirwan MRD (2021). Survey data of COVID-19 vaccine side effects among hospital staff in a national referral hospital in Indonesia. Data Brief.

[62] Riad A, Sağıroğlu D, Üstün B (2021). Prevalence and risk factors of CoronaVac side effects: an independent cross-sectional study among healthcare workers in Turkey. J Clin Med.

[63] Han B, Song Y, Li C (2021). Safety, tolerability, and immunogenicity of an inactivated SARS-CoV-2 vaccine (CoronaVac) in healthy children and adolescents: a double-blind, randomised, controlled, phase 1/2 clinical trial. Lancet Infect Dis.

[64] Jackson LA, Anderson EJ, Rouphael NG (2020). An mRNA vaccine against SARS-CoV-2 - preliminary report. N Engl J Med.

[65] Anderson EJ, Rouphael NG, Widge AT (2020). Safety and immunogenicity of SARS-CoV-2 mRNA-1273 vaccine in older adults. N Engl J Med.

[66] Heath PT, Galiza EP, Baxter DN (2021). Safety and efficacy of NVX-CoV2373 Covid-19 vaccine. N Engl J Med.

[67] Shinde V, Bhikha S, Hoosain Z (2021). Efficacy of NVX-CoV2373 Covid-19 vaccine against the B.1.351 variant. N Engl J Med.

[68] Keech C, Albert G, Cho I (2020). Phase 1–2 trial of a SARS-CoV-2 recombinant spike protein nanoparticle vaccine. N Engl J Med.

[69] Xia S, Zhang Y, Wang Y (2021). Safety and immunogenicity of an inactivated SARS-CoV-2 vaccine, BBIBP-CorV: a randomised, double-blind, placebo-controlled, phase 1/2 trial. Lancet Infect Dis.

[70] Guo W, Duan K, Zhang Y (2021). Safety and immunogenicity of an inactivated SARS-CoV-2 vaccine in healthy adults aged 18 years or older: a randomized, double-blind, placebo-controlled, phase 1/2 trial. EClinicalMedicine.

[71] Xia S, Duan K, Zhang Y (2020). Effect of an inactivated vaccine against SARS-CoV-2 on safety and immunogenicity outcomes: interim analysis of 2 randomized clinical trials. JAMA.

[72] Zhu FC, Guan XH, Li YH (2020). Immunogenicity and safety of a recombinant adenovirus type-5-vectored COVID-19 vaccine in healthy adults aged 18 years or older: a randomised, double-blind, placebo-controlled, phase 2 trial. Lancet.

[73] Zhu FC, Li YH, Guan XH (2020). Safety, tolerability, and immunogenicity of a recombinant adenovirus type-5 vectored COVID-19 vaccine: a dose-escalation, open-label, non-randomised, first-in-human trial. Lancet.

[74] Sadoff J, Gray G, Vandebosch A (2021). Safety and efficacy of single-dose Ad26.COV2.S vaccine against Covid-19. N Engl J Med.

[75] Pagotto V, Ferloni A, Mercedes Soriano M (2021). Active monitoring of early safety of Sputnik V vaccine in Buenos Aires. Argentina Medicina (B Aires).

[76] Menni C, Klaser K, May A (2021). Vaccine side-effects and SARS-CoV-2 infection after vaccination in users of the COVID symptom study app in the UK: a prospective observational study. Lancet Infect Dis.

[77] Al Kaabi N, Zhang Y, Xia S (2021). Effect of 2 inactivated SARS-CoV-2 vaccines on symptomatic COVID-19 infection in adults: a randomized clinical trial. JAMA.

[78] Shimabukuro TT, Kim SY, Myers TR (2021). Preliminary findings of mRNA Covid-19 vaccine safety in pregnant persons. N Engl J Med.

[79] Bae S, Lee YW, Lim SY (2021). Adverse reactions following the first dose of ChAdOx1 nCoV-19 vaccine and BNT162b2 vaccine for healthcare workers in South Korea. J Korean Med Sci.

[80] Park MJ, Choi YJ, Choi S (2021). Emergency department utilization by in-hospital healthcare workers after COVID-19 vaccination. J Korean Med Sci.

[81] Kim T, Park SY, Yu S (2021). Impacts of side effects to BNT162b2 and the first dose of ChAdOx1 Anti-SARS-CoV-2 vaccination on work productivity, the need for medical attention, and vaccine acceptance: a multicenter survey on healthcare workers in referral teaching hospitals in the Republic of Korea. Vaccines (Basel).

[82] McLaurin-Jiang S, Garner CD, Krutsch K (2021). Maternal and child symptoms following COVID-19 vaccination among breastfeeding mothers. Breastfeed Med.

[83] Song JY, Cheong HJ, Kim SR (2021). Early safety monitoring of COVID-19 vaccines in healthcare workers. J Korean Med Sci.

[84] Hatmal MM, Al-Hatamleh MAI, Olaimat AN (2021). Side effects and perceptions following COVID-19 vaccination in Jordan: a randomized, cross-sectional study implementing machine learning for predicting severity of side effects. Vaccines (Basel).

[85] Kim SH, Wi YM, Yun SY (2021). Adverse events in healthcare workers after the first dose of ChAdOx1 nCoV-19 or BNT162b2 mRNA COVID-19 vaccination: a single center experience. J Korean Med Sci.

[86] Powell AA, Power L, Westrop S (2021). Real-world data shows increased reactogenicity in adults after heterologous compared to homologous prime-boost COVID-19 vaccination, March-June 2021. England Euro Surveill.

[87] Al Khames Aga QA, Alkhaffaf WH, Hatem TH (2021). Safety of COVID-19 vaccines. J Med Virol.

[88] Jęśkowiak I, Wiatrak B, Grosman-Dziewiszek P (2021). The incidence and severity of post-vaccination reactions after vaccination against COVID-19. Vaccines (Basel).

[89] Shu YJ, He JF, Pei RJ (2021). Immunogenicity and safety of a recombinant fusion protein vaccine (V-01) against coronavirus disease 2019 in healthy adults: a randomized, double-blind, placebo-controlled, phase II trial. Chin Med J (Engl).

[90] Meng FY, Gao F, Jia SY (2021). Safety and immunogenicity of a recombinant COVID-19 vaccine (Sf9 cells) in healthy population aged 18 years or older: two single-center, randomised, double-blind, placebo-controlled, phase 1 and phase 2 trials. Signal Transduct Target Ther.

[91] Yang S, Li Y, Dai L (2021). Safety and immunogenicity of a recombinant tandem-repeat dimeric RBD-based protein subunit vaccine (ZF2001) against COVID-19 in adults: two randomised, double-blind, placebo-controlled, phase 1 and 2 trials. Lancet Infect Dis.

[92] Cherian S, Paul A, Ahmed S (2021). Safety of the ChAdOx1 nCoV-19 and the BBV152 vaccines in 724 patients with rheumatic diseases: a post-vaccination cross-sectional survey. Rheumatol Int.

[93] Andrzejczak-Grządko S, Czudy Z, Donderska M (2021). Side effects after COVID-19 vaccinations among residents of Poland. Eur Rev Med Pharmacol Sci.

[94] Ou MT, Boyarsky BJ, Chiang TPY (2021). Immunogenicity and reactogenicity after SARS-CoV-2 mRNA vaccination in kidney transplant recipients taking belatacept. Transplantation.

[95] Quiroga B, Sánchez-Álvarez E, Goicoechea M (2021). COVID-19 vaccination among Spanish nephrologists: acceptance and side effects. J Healthc Qual Res.

[96] Pan HX, Liu JK, Huang BY (2021). Immunogenicity and safety of a severe acute respiratory syndrome coronavirus 2 inactivated vaccine in healthy adults: randomized, double-blind, and placebo-controlled phase 1 and phase 2 clinical trials. Chin Med J (Engl).

[97] Alhazmi A, Alamer E, Daws D (2021). Evaluation of side effects associated with COVID-19 vaccines in Saudi Arabia. Vaccines (Basel).

[98] Abu-Hammad O, Alduraidi H, Abu-Hammad S (2021). Side effects reported by Jordanian healthcare workers who received COVID-19 vaccines. Vaccines (Basel).

[99] Wang J, Hou Z, Liu J (2021). Safety and immunogenicity of COVID-19 vaccination in patients with non-alcoholic fatty liver disease (CHESS2101): a multicenter study. J Hepatol.

[100] Campello E, Simion C, Bulato C (2021). Absence of hypercoagulability after nCoV-19 vaccination: an observational pilot study. Thromb Res.

[101] Zhang J, Hu Z, He J (2021). Safety and immunogenicity of a recombinant interferon-armed RBD dimer vaccine (V-01) for COVID-19 in healthy adults: a randomized, double-blind, placebo-controlled. Phase I trial Emerg Microbes Infect.

[102] Ward BJ, Gobeil P, Séguin A (2021). Phase 1 randomized trial of a plant-derived virus-like particle vaccine for COVID-19. Nat Med.

[103] Zdziarski K, Landowski M, Zabielska P (2021). Subjective feelings of polish doctors after receiving the COVID-19 vaccine. Int J Environ Res Public Health.

[104] Goepfert PA, Fu B, Chabanon AL (2021). Safety and immunogenicity of SARS-CoV-2 recombinant protein vaccine formulations in healthy adults: interim results of a randomised, placebo-controlled, phase 1–2, dose-ranging study. Lancet Infect Dis.

[105] Hwang YH, Song KH, Choi Y (2021). Can reactogenicity predict immunogenicity after COVID-19 vaccination?. Korean J Intern Med.

[106] Chappell KJ, Mordant FL, Li Z (2021). Safety and immunogenicity of an MF59-adjuvanted spike glycoprotein-clamp vaccine for SARS-CoV-2: a randomised, double-blind, placebo-controlled, phase 1 trial. Lancet Infect Dis.

[107] von Wrede R, Pukropski J, Moskau-Hartmann S (2021). COVID-19 vaccination in patients with epilepsy: first experiences in a German tertiary epilepsy center. Epilepsy Behav.

[108] Mulligan MJ, Lyke KE, Kitchin N (2020). Phase I/II study of COVID-19 RNA vaccine BNT162b1 in adults. Nature.

[109] Tebas P, Yang S, Boyer JD (2021). Safety and immunogenicity of INO-4800 DNA vaccine against SARS-CoV-2: a preliminary report of an open-label, Phase 1 clinical trial. EClinicalMedicine.

[110] Ruddy JA, Boyarsky BJ, Bailey JR (2021). Safety and antibody response to two-dose SARS-CoV-2 messenger RNA vaccination in persons with HIV. AIDS.

[111] García-Azorín D, Do TP, Gantenbein AR (2021). Delayed headache after COVID-19 vaccination: a red flag for vaccine induced cerebral venous thrombosis. J Headache Pain.

[112] Perrotta A, Biondi-Zoccai G, Saade W (2021). A snapshot global survey on side effects of COVID-19 vaccines among healthcare professionals and armed forces with a focus on headache. Panminerva Med.

[113] Ekizoglu E, Gezegen H, Yalınay Dikmen P (2021). The characteristics of COVID-19 vaccine-related headache: clues gathered from the healthcare personnel in the pandemic. Cephalalgia E-Pub.

[114] Hatmi ZN (2021). A systematic review of systematic reviews on the COVID-19 pandemic. SN Compr Clin Med.

[115] Leonardi M, Lee H, van der Veen S (2020). Avoiding the banality of evil in times of COVID-19: thinking differently with a biopsychosocial perspective for future health and social policies development. SN Compr Clin Med.

[116] Yu S, He M, Liu R (2013). Headache yesterday in China: a new approach to estimating the burden of headache, applied in a general-population survey in China. Cephalalgia.

[117] Andrée C, Steiner TJ, Barré J (2014). Headache yesterday in Europe. J Headache Pain.

[118] Ayzenberg I, Katsarava Z, Sborowski A (2015). Headache yesterday in Russia: its prevalence and impact, and their application in estimating the national burden attributable to headache disorders. J Headache Pain.

[119] Steiner TJ, Rao GN, Kulkarni GB (2016). Headache yesterday in Karnataka state, India: prevalence, impact and cost. J Headache Pain.

[120] Salih F, Schönborn L, Kohler S (2021). Vaccine-induced thrombocytopenia with severe headache. N Engl J Med.

[121] Göbel CH, Heinze A, Karstedt S (2021). Clinical characteristics of headache after vaccination against COVID-19 (coronavirus SARS-CoV-2) with the BNT162b2 mRNA vaccine: a multicentre observational cohort study. Brain Commun.

[122] Baig AM, Khaleeq A, Ali U (2020). Evidence of the COVID-19 virus targeting the CNS: tissue distribution, host–virus interaction, and proposed neurotropic mechanisms. ACS Chem Neurosci.

[123] Bougakov D, Podell K, Goldberg E (2021). Multiple Neuroinvasive Pathways in COVID-19. Mol Neurobiol.

[124] MaassenVanDenBrink A, de Vries T, Danser AHJ (2020). Headache medication and the COVID-19 pandemic. J Headache Pain.

[125] Wu Y, Xu X, Yang L (2020). Nervous system damage after COVID-19 infection: presence or absence?. Brain Behav Immun.

[126] Harapan BN, Yoo HJ (2021). Neurological symptoms, manifestations, and complications associated with severe acute respiratory syndrome coronavirus 2 (SARS-CoV-2) and coronavirus disease 19 (COVID-19). J Neurol.

[127] Caronna E, Pozo-Rosich P (2021). Headache as a symptom of COVID-19: narrative review of 1-year research. Curr Pain Headache Rep.

[128] Chou SH, Beghi E, Helbok R (2021). GCS-NeuroCOVID consortium and energy consortium. Global incidence of neurological manifestations among patients hospitalized with COVID-19 – a report for the GCS-NeuroCOVID consortium and the energy consortium. JAMA Netw Open.

[129] Borges do Nascimento IJ, Cacic N, Abdulazeem HM et al (2020) Novel coronavirus infection (COVID-19) in humans: a scoping review and meta-analysis. J Clin Med 9:941. 10.3390/jcm9040941 10.3390/jcm9040941 PMC7230636 32235486

[130] Lechien JR, Chiesa-Estomba CM, Place S (2020). Clinical and epidemiological characteristics of 1,420 European patients with mild-to-moderate coronavirus disease 2019. J Intern Med.

[131] Liguori C, Pierantozzi M, Spanetta M (2020). Subjective neurological symptoms frequently occur in patients with SARS-CoV2 infection. Brain Behav Immun.

[132] O’Keefe JB, Tong EJ, O’Keefe GD (2021). Description of symptom course in a telemedicine monitoring clinic for acute symptomatic COVID-19: a retrospective cohort study. BMJ Open.

[133] Straburzyński M, Nowaczewska M, Budrewicz S (2021). COVID-19-related headache and sinonasal inflammation: a longitudinal study analysing the role of acute rhinosinusitis and ICHD-3 classification difficulties in SARS-CoV-2 infection. Cephalalgia.

[134] Bolay H, Gül A, Baykan B (2020). COVID-19 is a real headache!. Headache.

[135] Bobker SM, Robbins MS (2021). Virtual issue: COVID-19 and headache. Headache.

[136] Messlinger K, Neuhuber W, May A (2021). Activation of the trigeminal system as a likely target of SARS-CoV-2 may contribute to anosmia in COVID-19. Cephalalgia.

[137] Collignon C, Bol V, Chalon A (2020). Innate immune responses to chimpanzee adenovirus vector 155 vaccination in mice and monkeys. Front Immunol.

[138] Bergamaschi C, Terpos E, Rosati M (2021). Systemic IL-15, IFN-γ, and IP-10/CXCL10 signature associated with effective immune response to SARS-CoV-2 in BNT162b2 mRNA vaccine recipients. Cell Rep.

[139] Pépin S, Donazzolo Y, Jambrecina A (2013). Safety and immunogenicity of a quadrivalent inactivated influenza vaccine in adults. Vaccine.

[140] Trigo López J, García-Azorín D, Planchuelo-Gómez Á (2020). Phenotypic characterization of acute headache attributed to SARS-CoV-2: an ICHD-3 validation study on 106 hospitalized patients. Cephalalgia.

[141] Wang YH, Huang LY, Chen YL (2021). ChAdOx1 COVID-19 vaccine-induced thrombocytopenia syndrome. QJM.

[142] Giovane R, Campbell J (2021). Bilateral thalamic stroke: a case of COVID-19 vaccine-induced immune thrombotic thrombocytopenia (VITT) or a coincidence due to underlying risk factors?. Cureus.

