# Supplementary material for: Headache onset after vaccination against SARS-CoV-2: a systematic literature review and meta-analysis
Source: J Headache Pain. 2022 Mar 31;23(1):41. doi: 10.1186/s10194-022-01400-4 (PMC8969402; doi:10.1186/s10194-022-01400-4)
Supplement: Supplementary file 1 — Additional file 1. [file 10194_2022_1400_MOESM1_ESM.docx]

**Supplementary Materials**

Castaldo M, Waliszewska-Prosół M, Koutsokera M, Robotti M, Straburzyński M, Apostolakopoulou L, Capizzi M, Çibuku O, Ambat FDF, Frattale I, Gadzhieva Z, Gallo E, Gryglas-Dworak A, Halili G, Jusupova A, Koperskaya Y, Leheste AL, Manzo ML, Marcinnò A, Marino A, Mikulenka P, Ong BE, Polat B, Popovic Z, Rivera Mancilla E, Roceanu AM, Rollo E, Romozzi M, Ruscitto C, Scotto di Clemente F, Strauss S, Taranta V, Terhart M, Tychenko I, Vigneri S, Misiak B, Martelletti P, Raggi A. **Headache onset after vaccination against SARS-CoV-2: a systematic literature review and meta-analysis**. The Journal of Headache and Pain

**Search Strings**

**1) EMBASE Synthesis**

[Coronarvirus OR SARS-CoV2 (emtree) OR COVID (free search in ti/abs)] AND [SARS-CoV2 Vaccine (emtree) OR Vaccin (free search in ti/abs)] AND [headache (emtree) OR headache (free search in ti/abs) OR trial OR side effect (free search in ti/abs)]. Limits: records published in 2020-2021, in English, with an Abstract, on EMBASE only.

**2) EMBASE Full search string**

('coronavirus disease 2019'/exp OR 'severe acute respiratory syndrome coronavirus 2'/exp OR coronavirus:ab,ti OR 'covid 19':ab,ti OR 'covid or 2019-ncov':ab,ti OR 'hcov 19':ab,ti OR 'sars cov 2':ab,ti OR 'sars cov':ab,ti OR 'severe acute respiratory syndrome':ab,ti OR 'spike protein':ab,ti) AND ('sars-cov-2 vaccine'/exp OR 'vaccin*' OR 'antibody response' OR 'antiviral therapy' OR 'neutralising antibodies' OR 'immunity' OR 'immunization' OR 'booster' OR 'injection' OR 'inoculation' OR 'virus-based anti-covid' OR 'pfizer' OR 'pfizer-biontech' OR 'fosun pharma' OR 'bnt162b1' OR 'tozinameran' OR 'bnt162b2' OR 'comirnaty' OR 'mrna-based' OR 'moderna' OR 'mrna-1273' OR 'cx-024414' OR 'niaid' OR 'elasomeran' OR 'spikevax' OR 'mrna-1283' OR 'ad26covs1or' OR 'janssen covid-19 vaccine' OR 'johnson & johnson' OR 'vac31518' OR 'jnj-78436735' OR 'sputnik v' OR 'gamaleya sputnik v' OR 'gam-covid-vac-lyo' OR 'rad26 vector-based' OR 'rad5 vector-based' OR 'ad26 vector' OR 'ad26.cov2.s' OR 'ad26covs1' OR 'biocad' OR 'oxford' OR 'astrazeneca' OR 'vaxzevria' OR 'azd1222' OR 'chadox1' OR 'covishied' OR 'sinovac' OR 'coronavac' OR 'bbibp-corv' OR 'bibp' OR 'wibp-corv' OR 'sinopharm' OR 'novavax' OR 'recombinant protein nanoparticles' OR 'nvx-cov2372' OR 'covaxin' OR 'bbv152' OR 'bharat biotech' OR 'gamaleya research institute' OR 'wuhan institute of biological products' OR 'beijing institute of biological products' OR 'chinese academy of medical sciences' OR 'chumakov federal scientific center' OR 'adenovirus' OR 'convidecia epivac' OR 'cansino' OR 'corona vector institute' OR 'zf2001' OR 'rbd-dimer' OR 'zifivax' OR 'abdala' OR 'cigb-66' OR 'kconvac' OR 'coviran barakat' OR 'finlay-fr-2' OR 'picovacc' OR 'covivac' OR 'qazcovid-in' OR 'ad5-ncov' OR 'cvncov' OR 'minhai' OR 'imbcams' OR 'qazcovac-p' OR 's-only' OR 'pakvac' OR 'grad-cov2' OR 'epivaccorona' OR 'reithera' OR 'curevac ag') AND ('headache'/exp OR 'headache*' OR 'migraine' OR 'cephalalgia*' OR 'hemicrania' OR 'head pain' OR 'neuralgia' OR 'ndph' OR 'tth' OR 'ch' OR 'cranial pain' OR 'cephalgia*' OR 'random*' OR 'blind*' OR 'phase iii' OR 'phase 3' OR 'phase iv' OR 'phase 4' OR 'phase 2/3' OR 'phase ii/iii' OR 'case-control' OR 'case control' OR 'controlled trial' OR 'controlled stud*' OR 'clinical trial' OR 'placebo*' OR 'rct' OR 'cross-sectional' OR 'cross sectional' OR 'regist*' OR 'longitudinal' OR 'cohort' OR 'panel stud*' OR 'real-life' OR 'real life' OR 'real world' OR 'real-world' OR 'open' OR 'prospect*' OR 'observational' OR 'transverse' OR 'side effect*' OR 'adverse event*' OR 'adverse effect*' OR 'ae*' OR 'sae*' OR 'adverse response*' OR 'adverse reaction*' OR 'safe*' OR 'reactogenicity') NOT ([medline]/lim OR [pubmed-not-medline]/lim) AND [english]/lim AND [abstracts]/lim AND [2020-2021]/py

**3) PubMed Synthesis**

[COVID-19 (MeSH) OR COVID (free search in ti/abs)] AND [COVID-19 Vaccines (MeSH) OR Vaccin (free search in ti/abs)] AND [headache (MeSH) OR headache (free search in ti/abs) OR trial OR side effect (free search in ti/abs)]. Limits: records published in 2020-2021, in English, with an Abstract

**4) PubMed Full search string**

((""COVID-19""[MeSH Terms] OR (""Coronavirus""[Title/Abstract] OR ""COVID-19""[Title/Abstract] OR ""COVID""[Title/Abstract] OR ""2019-nCoV""[Title/Abstract] OR ""hcov 19""[Title/Abstract] OR ""hcov 19""[Title/Abstract] OR ""SARS-CoV-2""[Title/Abstract] OR ""SARS-CoV""[Title/Abstract] OR ""Severe acute respiratory syndrome""[Title/Abstract] OR ""Spike protein""[Title/Abstract])) AND (""COVID-19 Vaccines""[MeSH Terms] OR (""vaccin*""[Title/Abstract] OR ""Antibody response""[Title/Abstract] OR ""Antiviral therapy""[Title/Abstract] OR ""Neutralising antibodies""[Title/Abstract] OR ""Immunity""[Title/Abstract] OR ""Immunization""[Title/Abstract] OR ""Booster""[Title/Abstract] OR ""Injection""[Title/Abstract] OR ""Inoculation""[Title/Abstract] OR ""Adenovirus""[Title/Abstract] OR ""Pfizer""[Title/Abstract] OR ""Pfizer-BioNTech""[Title/Abstract] OR ""BNT162b1""[Title/Abstract] OR ""Tozinameran""[Title/Abstract] OR ""BNT162b2""[Title/Abstract] OR ""Comirnaty""[Title/Abstract] OR ""mRNA-based""[Title/Abstract] OR ""Moderna""[Title/Abstract] OR ""mRNA-1273""[Title/Abstract] OR ""NIAID""[Title/Abstract] OR ""Janssen Covid-19 Vaccine""[Title/Abstract] OR ""johnson johnson""[Title/Abstract] OR ""JNJ-78436735""[Title/Abstract] OR ""Sputnik V""[Title/Abstract] OR ""Ad26 vector""[Title/Abstract] OR ""Ad26.COV2.S""[Title/Abstract] OR ""Biocad""[Title/Abstract] OR ""Oxford""[Title/Abstract] OR ""AstraZeneca""[Title/Abstract] OR ""Vaxzevria""[Title/Abstract] OR ""AZD1222""[Title/Abstract] OR ""ChAdOx1""[Title/Abstract] OR ""SinoVac""[Title/Abstract] OR ""CoronaVac""[Title/Abstract] OR ""BBIBP-CorV""[Title/Abstract] OR ""BIBP""[Title/Abstract] OR ""Sinopharm""[Title/Abstract] OR ""Novavax""[Title/Abstract] OR ""Covaxin""[Title/Abstract] OR ""BBV152""[Title/Abstract] OR ""Bharat Biotech""[Title/Abstract] OR ""Gamaleya Research Institute""[Title/Abstract] OR ""Chinese Academy of Medical Sciences""[Title/Abstract] OR ""Chumakov Federal Scientific center""[Title/Abstract] OR ""Adenovirus""[Title/Abstract] OR ""CanSino""[Title/Abstract] OR ""ZF2001""[Title/Abstract] OR ""RBD-Dimer""[Title/Abstract] OR ""Abdala""[Title/Abstract] OR ""KCONVAC""[Title/Abstract] OR ""PiCoVacc""[Title/Abstract] OR ""CoviVac""[Title/Abstract] OR ""Ad5-nCoV""[Title/Abstract] OR ""CvnCoV""[Title/Abstract] OR ""Minhai""[Title/Abstract] OR ""S-only""[Title/Abstract] OR ""GRAd-COV2""[Title/Abstract] OR ""EpiVacCorona""[Title/Abstract] OR ""CureVac AG""[Title/Abstract])) AND (""Headache""[MeSH Terms] OR (""headache*""[Title/Abstract] OR ""Migraine""[Title/Abstract] OR ""cephalalgia*""[Title/Abstract] OR ""Hemicrania""[Title/Abstract] OR ""Head pain""[Title/Abstract] OR ""Neuralgia""[Title/Abstract] OR ""NDPH""[Title/Abstract] OR ""TTH""[Title/Abstract] OR ""CH""[Title/Abstract] OR ""Cranial Pain""[Title/Abstract] OR ""cephalgia*""[Title/Abstract]) OR (""random*""[Title/Abstract] OR ""blind*""[Title/Abstract] OR ""phase III""[Title/Abstract] OR ""phase 3""[Title/Abstract] OR ""phase IV""[Title/Abstract] OR ""phase 4""[Title/Abstract] OR ""phase 2/3""[Title/Abstract] OR ""phase II/III""[Title/Abstract] OR ""case-control""[Title/Abstract] OR ""case-control""[Title/Abstract] OR ""controlled trial""[Title/Abstract] OR ""controlled stud*""[Title/Abstract] OR ""clinical trial""[Title/Abstract] OR ""placebo*""[Title/Abstract] OR ""RCT""[Title/Abstract] OR ""cross-sectional""[Title/Abstract] OR ""cross-sectional""[Title/Abstract] OR ""regist*""[Title/Abstract] OR ""longitudinal""[Title/Abstract] OR ""cohort*""[Title/Abstract] OR ""panel stud*""[Title/Abstract] OR ""real-life""[Title/Abstract] OR ""real-life""[Title/Abstract] OR ""real-world""[Title/Abstract] OR ""real-world""[Title/Abstract] OR ""open""[Title/Abstract] OR ""prospect*""[Title/Abstract] OR ""observational""[Title/Abstract] OR ""transverse""[Title/Abstract] OR ""side effect*""[Title/Abstract] OR ""adverse event*""[Title/Abstract] OR ""adverse effect*""[Title/Abstract] OR ""ae""[Title/Abstract] OR ""sae""[Title/Abstract] OR ""adverse response*""[Title/Abstract] OR ""adverse reaction*""[Title/Abstract] OR ""safe*""[Title/Abstract] OR ""reactogenicity""[Title/Abstract]))) AND ((fha[Filter]) AND (english[Filter]) AND (2020:2021[pdat]))

**Forest Plots**

**Figure S1: Forest plot for headache onset after injection, all vaccines together, first dose**

**Figure S2: Forest plot for headache onset after injection, all vaccines together, second dose**

**Figure S3: Forest plot for headache onset after injection, placebo recipients, first dose**

**Figure S4: Forest plot for headache onset after injection, placebo recipients, second dose**

**Figure S5: Forest plot for headache onset after injection, mRNA vaccine recipients, first dose**

**Figure S6: Forest plot for headache onset after injection, traditional vaccine recipients, first dose**

**Figure S7: Forest plot for headache onset after injection, BNT162b2 recipients, first dose**

**Figure S8: Forest plot for headache onset after injection, BNT162b2 recipients, second dose**

**Figure S9: Forest plot for headache onset after injection, ChAdOx1 recipients, first dose**

**Figure S10: Forest plot for headache onset after injection, ChAdOx1 recipients, second dose**

**Figure S11: Forest plot for headache onset after injection, mRNA-1273 recipients, first dose**

**Figure S12: Forest plot for headache onset after injection, mRNA-1273 recipients, second dose**

**Figure S13: Forest plot for headache onset after injection, NVX-CoV2373 recipients, first dose**

**Figure S14: Forest plot for headache onset after injection, NVX-CoV2373 recipients, second dose**

**Figure S15: Forest plot for headache onset after injection, PiCoVacc recipients, first dose**
